# Targeting the chemerin/CMKLR1 axis by small molecule antagonist α-NETA mitigates endometriosis progression

**DOI:** 10.3389/fphar.2022.985618

**Published:** 2022-11-29

**Authors:** Ming Yu, Yali Yang, Hao Zhao, Mengxia Li, Jie Chen, Baobei Wang, Tianxia Xiao, Chen Huang, Huashan Zhao, Wei Zhou, Jian V. Zhang

**Affiliations:** ^1^ Center for Energy Metabolism and Reproduction, Shenzhen Institute of Advanced Technology, Chinese Academy of Sciences, Shenzhen, China; ^2^ Shenzhen Institute of Advanced Technology, Chinese Academy of Sciences, Shenzhen, China; ^3^ Shenzhen Key Laboratory of Metabolic Health, Shenzhen, China; ^4^ Shenzhen College of Advanced Technology, University of Chinese Academy of Sciences, Shenzhen, China; ^5^ College of Life Science, Northeast Forestry University, Harbin, China; ^6^ Huazhong University of Science and Technology Union Shenzhen Hospital, Shenzhen, China

**Keywords:** endometriosis, chemerin, CMKLR1, small molecule antagonist, α-NETA

## Abstract

Endometriosis is a common gynecological disease, characterized by the presence of endometrial-like lesions outside the uterus. This debilitating disease causes chronic pelvic pain and infertility with limited therapeutics. Chemerin is a secretory protein that acts on CMKLR1 (Chemokine-Like Receptor 1) to execute functions vital for immunity, adiposity, and metabolism. Abnormal chemerin/CMKLR1 axis underlies the pathological mechanisms of certain diseases including cancer and inflammatory diseases, but its role in endometriosis remains unknown. Herein, our results showed that chemerin and CMKLR1 are up-regulated in endometriotic lesions by analyzing the human endometriosis database and murine model. Knockdown of chemerin or CMKLR1 by shRNA led to mesenchymal-epithelial transition (MET) along with compromised viability, migration, and invasion of hEM15A cells. Most importantly, 2-(α-naphthoyl) ethyltrimethylammonium iodide (α-NETA), a small molecule antagonist for CMKLR1, was evidenced to exhibit profound anti-endometriosis effects (anti-growth, anti-mesenchymal features, anti-angiogenesis, and anti-inflammation) *in vitro* and *in vivo*. Mechanistically, α-NETA exhibited a dual inhibition effect on PI3K/Akt and MAPK/ERK signaling pathways in hEM15A cells and murine endometriotic grafts. This study highlights that the chemerin/CMKLR1 signaling axis is critical for endometriosis progression, and targeting this axis by α-NETA may provide new options for therapeutic intervention.

## Introduction

Endometriosis is a common gynecological disease estimated to affect 6–10% of women of reproductive age (Zondervan et al., 2020). It is characterized by the presence of endometrial-like lesions outside the uterus, and could be divided into three sub-types based on its histopathology and anatomical locations: superficial peritoneal endometriosis, ovarian endometriosis (also known as endometrioma or chocolate cyst), and deep infiltrating endometriosis (DIE) (nodules >5 mm in depth) (Wang et al., 2020). Notably, this heterogeneous disease is closely associated with chronic pelvic pain and infertility, which severely affect the living quality of women and cause the socioeconomic burden (Taylor et al., 2021). Several theories were proposed to explain the origin and pathophysiology of endometriosis, and at least for peritoneal endometriosis, Sampson’s “retrograde menstruation” theory has been widely accepted (Zondervan et al., 2018). It refers that menstrual endometrial debris reaches the abdominal cavity through the fallopian tubes, where they undergo successive biological events (adhesion, survival, proliferation, invasion, angiogenesis, inflammation, etc.) to form the endometriotic lesions (Zondervan et al., 2018). Advances made during the last 2 decades have unveiled that numerous factors (inheritance, genetic mutations, hormone metabolism, chronic inflammation, neoangiogenesis, etc.) account for the disease occurrence, progression, and performance (Saunders and Horne, 2021). Unfortunately, reliable non-invasive biomarkers for early diagnosis are still lacking and first-line treatments (surgery and medication management) for endometriosis are sub-optimal (Chapron et al., 2019). Therefore, identifying novel molecular targets and understanding the mechanism of endometriosis development are urgently needed to improve diagnostics and therapeutic methods.

The chemerin gene, *RARRES2* (retinoic acid receptor responder 2) was originally identified as a novel retinoid-responsive gene in skin (Nagpal et al., 1997). In 2003, two independent groups reported that chemerin, the product of *RARRES2*, was the endogenous ligand for an orphan G-protein coupled receptor (GPCR): chemokine-like receptor 1 (CMKLR1) (Meder et al., 2003; Wittamer et al., 2003). In the beginning, chemerin was mainly treated as a chemokine since it recruits the CMKLR1-expressing leukocytes to the inflammatory site to regulate the immune events (Wittamer et al., 2003), indicating the pro-inflammatory property of chemerin similar to other chemokines. Apart from the well-documented role in immunity, the chemerin/CMKLR1 axis was found to participate in numerous physiological processes including adipogenesis, angiogenesis, thermogenesis, and metabolism (Goralski et al., 2007; Bozaoglu et al., 2010; Takahashi et al., 2011; Lin et al., 2021). So far, studies also demonstrated that chemerin binds G protein-coupled receptor 1 (GPR1) and chemokine (C-C motif) receptor-like 2 (CCRL2) with a high affinity similar to that of CMKLR1. CMKLR1 displays a strong signals response to chemerin (intracellular Ca^2+^ release, inhibition of cAMP accumulation, and phosphorylation of ERK1/2). Unlike CMKLR1, the binding of chemerin to GPR1 results in a weak Ca^2+^ mobilization and phosphorylation of ERK1/2, and the binding of chemerin to CCRL2 does not signal nor internalize (De Henau et al., 2016). The distinct signaling properties of the chemerin receptors imply that CMKLR1 serves as the main functional receptor.

Abnormal chemerin/CMKLR1 axis closely correlates with several diseases including inflammatory diseases (rheumatoid arthritis, lupus nephritis, etc.), metabolic disorders (obesity, diabetes, etc.), cardiovascular diseases, gestational complications (preeclampsia, gestational diabetes mellitus, etc.), and cancer (Rourke et al., 2013; Goralski et al., 2019; Gutaj et al., 2020). Chemerin/CMKLR1 axis regulates numerous pathogenetic events (migration, invasion, angiogenesis, immune cell infiltration, etc.) to further promote the disease progression. Noteworthy, 2-(α-naphthoyl) ethyltrimethylammonium iodide (α-NETA), a small molecule antagonist for CMKLR1 was identified (Graham et al., 2014), and this compound has shown promising therapeutic effects in various disease models (Graham et al., 2014; Tümmler et al., 2017; Neves et al., 2018). In 2015, literature reported that chemerin and CMKLR1 expression were aberrantly up-regulated in the ovarian endometrioma tissues relative to the eutopic endometrial tissues (Jin et al., 2015), but the pathological mechanisms by which the chemerin/CMKLR1 axis modulates endometriosis remain lacking. Accordingly, herein, we endeavored to study the role of the chemerin/CMKLR1 axis in endometriosis and to access whether antagonizing this axis by α-NETA could ameliorate the endometriosis progression.

## Results

### Chemerin and CMKLR1 are up-regulated in endometriosis

**FIGURE 1:**
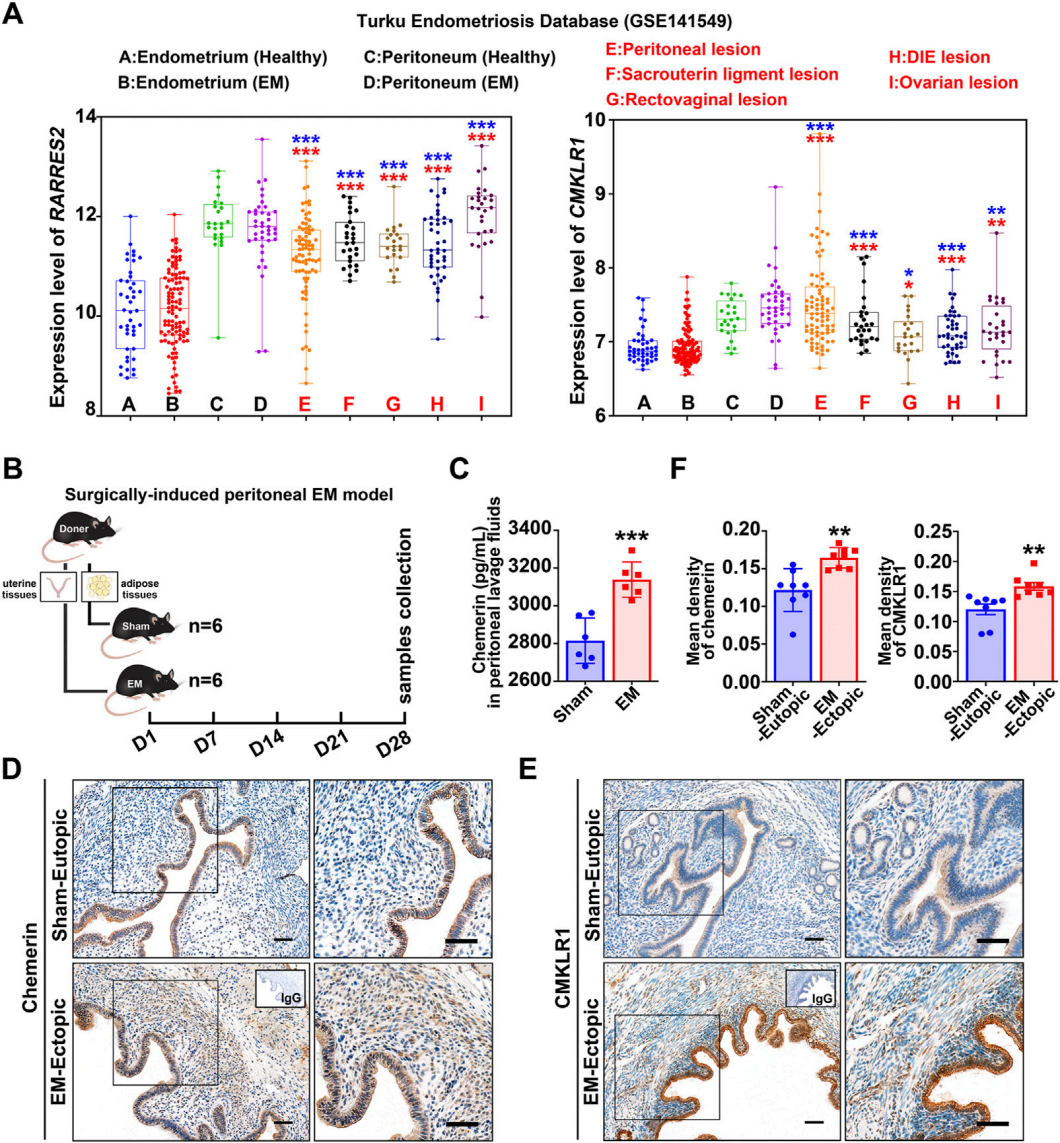
Chemerin and CMKLR1 are up-regulated in endometriosis. **(A)** Chemerin and CMKLR1 transcript levels in the endometrium, peritoneum, and endometriotic lesions from Turku endometriosis database (GSE141549). ns, not significant; blue, *versus* healthy endometrium; red, *versus* endometriosis endometrium; **(B)** Schematic of animal experimental-1 to analyze the chemerin expression in a surgically-induced peritoneal EM mouse model. **(C)** After surgeries for 1 month, chemerin levels in peritoneal lavage fluids from sham- (*n* = 6) and EM-mice (*n* = 6) were detected by ELISA. **(D**,**E)** Representative IHC images of murine chemerin and CMKLR1 expression in sham-eutopic endometrium and EM-ectopic lesions. Bars, 100 μm. **(F)** The quantitative data of IHC in **(D**,**E)** was shown. Data are shown as mean ± SD; *p < 0.05; **p < 0.01; ***p < 0.001.

To detect the expression of chemerin and CMKLR1 in human endometriosis, we analyzed the transcript data in the Turku endometriosis database (https://endometdb.utu.fi/) (GEO accession: GSE141549), and results showed that transcript levels for chemerin and CMKLR1 in endometriotic tissues from five locations (peritoneal, sacrouterine ligament, rectovaginal, DIE, and ovarian lesions) were consistently enhanced compared to the healthy endometrium (Figure 1A). Next, we established a murine peritoneal endometriosis model to confirm this finding (Figure 1B), and ELISA results showed that chemerin was significantly enhanced in peritoneal lavage fluids of EM mice (3131.6 ± 102.7 pg/ml) compared with those of sham-treated mice (2814.9 ± 120.2 pg/ml) (Figure 1C). IHC was performed to verify the immunolocalization of chemerin and CMKLR1, and as depicted in Figure 1D, chemerin signals were confined to the glandular epithelium of sham-eutopic uterus, while almost invisible in the stroma. Notably, chemerin staining was observed both in the glandular epithelium and the stroma of EM-lesions. Meanwhile, positive signals for CMKLR1 were localized to the glandular epithelial cells, stromal cells and vascular epithelial cells in the murine endometriotic grafts (Figure 1E; Supplementary Figure S1A). Quantitative data further showed that chemerin and CMKLR1 protein level were significantly elevated in the EM-lesions relative to the sham group (Figure 1F). These results provided evidence that chemerin and CMKLR1 expression were abnormally up-regulated in endometriosis.

### Knockdown of chemerin or CMKLR1 impairs the viability and mesenchymal features of hEM15A cells

**FIGURE 2:**
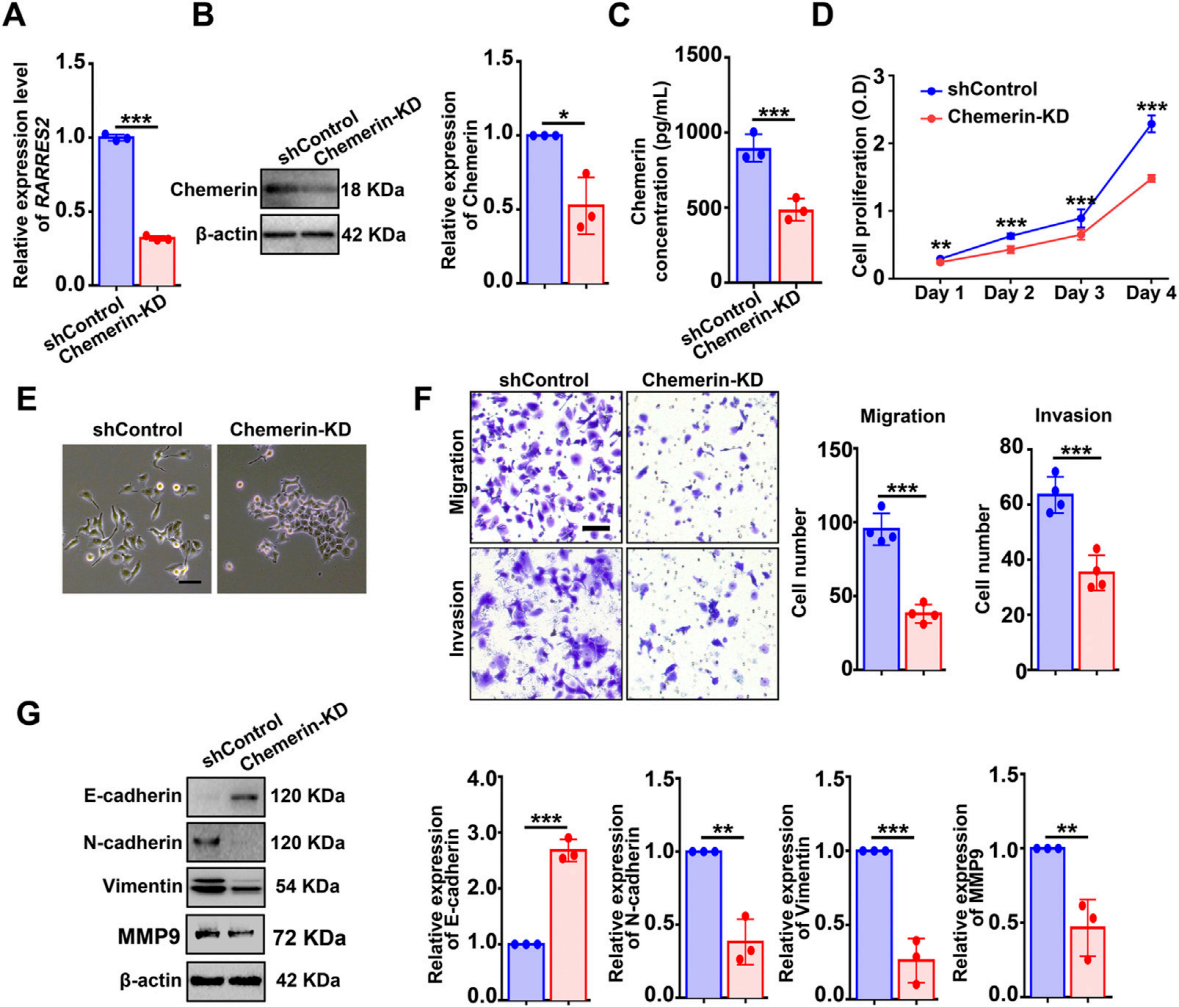
Knockdown of chemerin impairs the viability and mesenchymal features of hEM15A cells. **(A–C)** Chemerin mRNA and protein levels in hEM15A cells stably expressed scrambled shRNA (shControl) and shRNA targeting chemerin (chemerin-KD) were measured by qPCR, western blot, and ELISA. **(D)** CCK-8 assay was used to access the cell viability. **(E)** Representative images of cellular morphology. Scale Bars, 50 μm. **(F)** Transwell assay was utilized to evaluate cell migration and invasion ability, and the quantitative data was shown in the diagrams. **(G)** E-cadherin, N-cadherin, vimentin, and MMP-9 expression was detected by immunoblots. Assays were performed in triplicate and the densitometry of blots was shown. β-Actin served as a loading control. Data are shown as mean ± SD. *p < 0.05; **p < 0.01; ***p < 0.001.

To evaluate the function of chemerin in cellular biological events, hEM15A cells (an immortalized endometrial stromal cell line derived from eutopic endometrium of ovarian endometriosis (Mai et al., 2021)) were utilized*.* First, hEM15A and Ishikawa (a human endometrial adenocarcinoma cell line) cells were found to contain detectable chemerin protein as measured by western blot (Supplementary Figure S1B). Next, a single cell-clone line of hEM15A stably expressing shRNA targeting *RARRES2* was created. qPCR, Western blot, and ELISA were performed to validate the knockdown efficiency (Figures 2A–C). CCK-8 assay showed that chemerin knockdown (chemerin-KD) markedly suppressed the cell viability when compared with the shControl (Figure 2D). Additionally, we observed that shControl cells exhibited a typical mesenchymal-like morphology (loss of cell-cell contacts and cell scattering), while chemerin-KD cells displayed an epithelial-like morphology (gain of cell-cell contacts and cobblestone-like appearance) (Figure 2E). This mesenchymal-epithelial transformation (MET) of cellular morphology implies that chemerin-KD cells might lose the characteristics of mesenchymal cells such as the highly motility and invasiveness. As expected, transwell analysis results showed that the migratory and invasive capacities of chemerin-KD cells were markedly inhibited by ∼60% and ∼40%, respectively, when compared with the shControl cells (Figure 2F). Western blot results further showed that knockdown of endogenous chemerin led to up-regulated expression of E-cadherin (epithelial cell biomarker), in conjunction with down-regulated expression of N-cadherin, vimentin, and MMP9 (mesenchymal cell biomarkers) (Figure 2G), clearly suggesting the molecular alterations related to MET.

**FIGURE 3:**
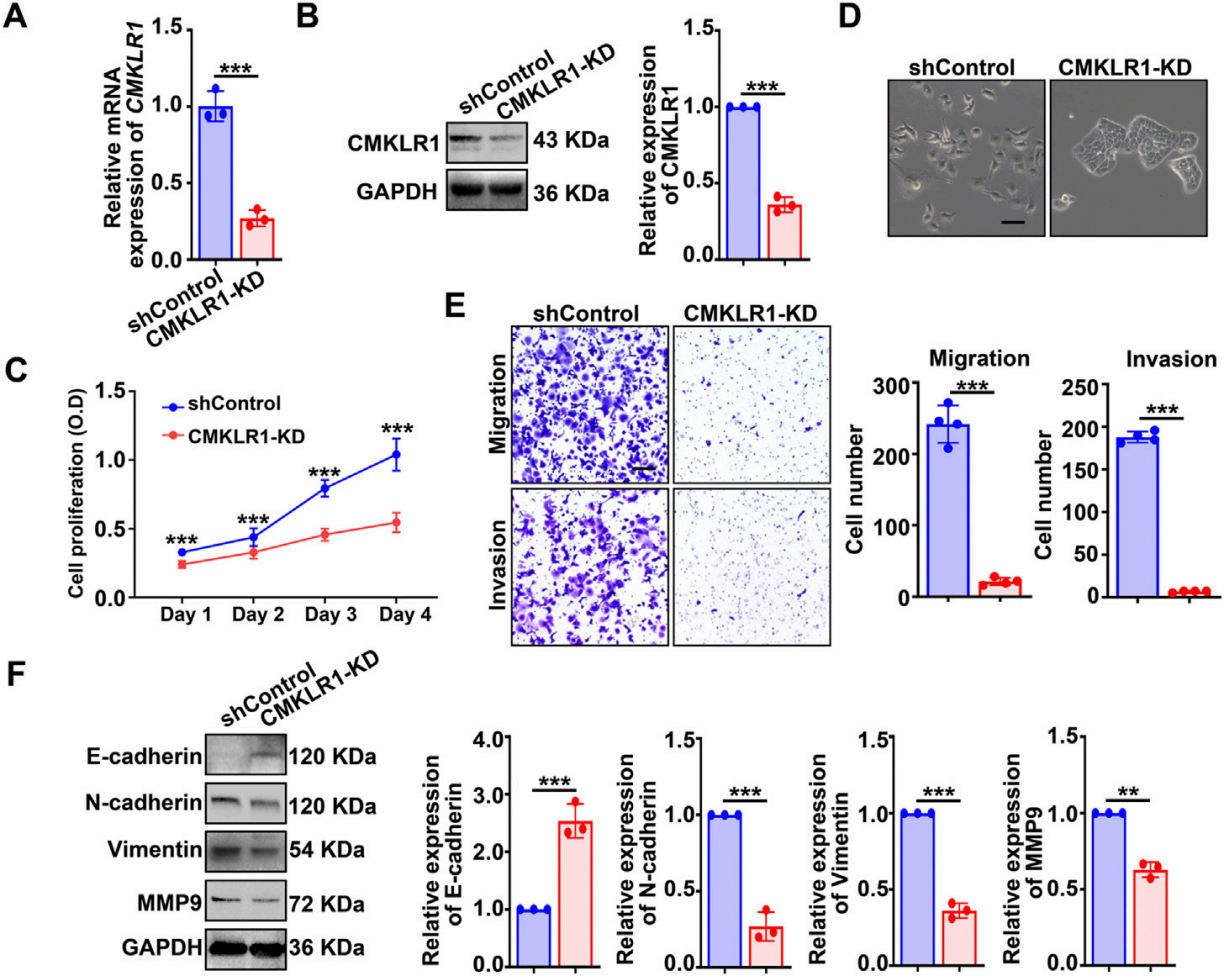
Knockdown of CMKLR1 impairs the viability and mesenchymal features of hEM15A cells. **(A,B)** CMKLR1 mRNA and protein levels in hEM15A cells stably expressed scrambled shRNA (shControl) and shRNA targeting CMKLR1 (CMKLR1-KD) were measured by qPCR and western blot. **(C)** CCK-8 assay was used to access the cell viability. **(D)** Representative images of cellular morphology. Scale Bars, 50 μm. **(E)** Transwell assay was utilized to evaluate cell migration and invasion ability, and the quantitative data was shown in the diagrams. **(F)** E-cadherin, N-cadherin, vimentin, and MMP-9 expression was detected by immunoblots. Assays were performed in triplicate and the densitometry of blots was shown. GAPDH served as a loading control. Data are shown as mean ± SD. *p < 0.05; **p < 0.01; ***p < 0.001.

Moving forward, we established a single cell-clone line of hEM15A stably expressing shRNA targeting *CMKLR1* (Figures 3A,B). Consistent with the findings found in chemerin-KD cells, CMKLR1-KD cells also exhibited an epithelial-like morphology (Figure 3C), compromised cellular viability (Figure 3D) as well as weakened motility and invasiveness (Figure 3E). Knockdown of CMKLR1 resulted in enhanced expression of E-cadherin, together with reduced expression of N-cadherin, vimentin, and MMP9 (Figure 3F). These data suggest that chemerin derived from endometriotic stromal cells serves as a critical modulator in sustaining the cellular viability and mesenchymal features *via* CMKLR1 in an autocrine feature.

### α-NETA inhibits the viability and mesenchymal features of hEM15A cells

**FIGURE 4:**
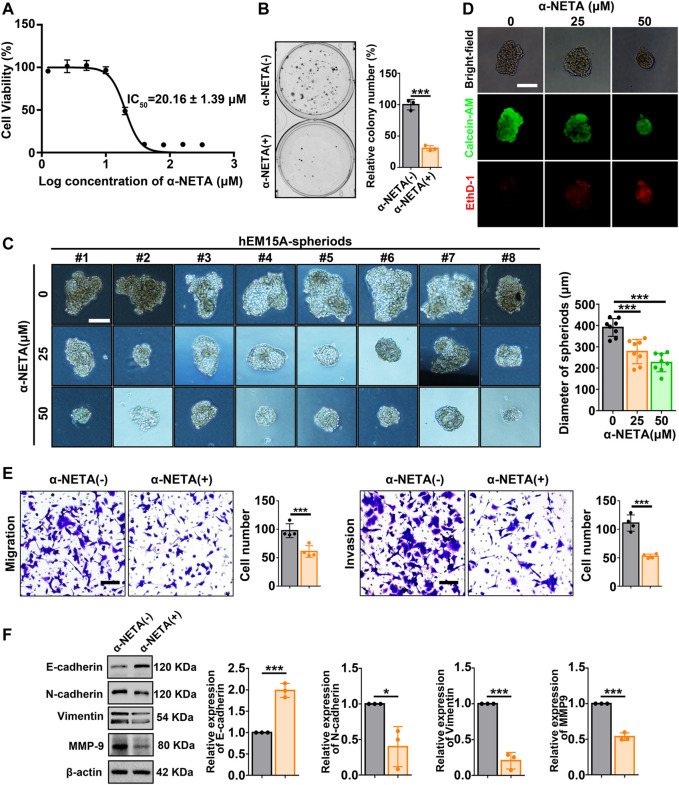
Effects of α-NETA on hEM15A-spheroids and hEM15A cells. **(A)** hEM15A cells were exposed to increasing concentrations of α-NETA for 24 h, a CCK-8 assay was used to access the cell viability, and the IC_50_ was calculated. **(B)** Clone formation assay of hEM15A cells treated without or with 25 μM α-NETA. **(C)** Representative images of hEM15A-spheriods treated with α-NETA (25 and 50 μM) for 48 h. The diameter of spheroids was recorded, and the statistical data was shown in the diagram. **(D)** Representative images of hEM15A-spheriods stained with calcein-AM and EthD-1 after α-NETA treatment. **(E)** Transwell assay was utilized to evaluate cell migration and invasion ability, and the quantitative data was shown in the diagrams. **(F)** E-cadherin, N-cadherin, vimentin, and MMP-9 expression were detected by immunoblots. Assays were performed in triplicate and the densitometry of blots was shown. β-Actin served as a loading control. Scale bars, 50 μM **(C,D)** and 100 μm **(E)**. Data are shown as mean ± SD. *p < 0.05; **p < 0.01; ***p < 0.001.

Next, a pharmacological approach was performed by utilizing the small molecule α-NETA to antagonize CMKLR1. CCK-8 assay showed that hEM15A cells displayed restrained viability with increasing concentrations of α-NETA exposure, and the IC_50_ value (20.16 ± 1.39 μm) was calculated (Figure 4A). Clone formation assay further observed that the clonogenic ability of hEM15A cells was inhibited by approximately 70% when the culture systems were persistently treated with 25 μM α-NETA (Figure 4B). Moreover, under the 3D-culture condition, α-NETA (25 and 50 μM) inhibited the growth of hEM15A-spheroids as measured by the diameters (Figure 4C) as well as stained with the calcein-AM (CaAM, living cell marker) and ethidium homodimer-1 (EthD-1, dead cell marker) (Figure 4D). Migration and invasion of hEM15A cells exposed to α-NETA were significantly decreased by around 40% and 50% relative to the controls, respectively (Figure 4E). Western blot results further verified that α-NETA treatment significantly altered the expression of biomarkers (E-cadherin, N-cadherin, vimentin, and MMP9) indicative of MET (Figure 4F).

### Chemerin acts on CMKLR1 to activate the PI3K/Akt and MAPK/ERK signaling pathways in hEM15A cells

**FIGURE 5:**
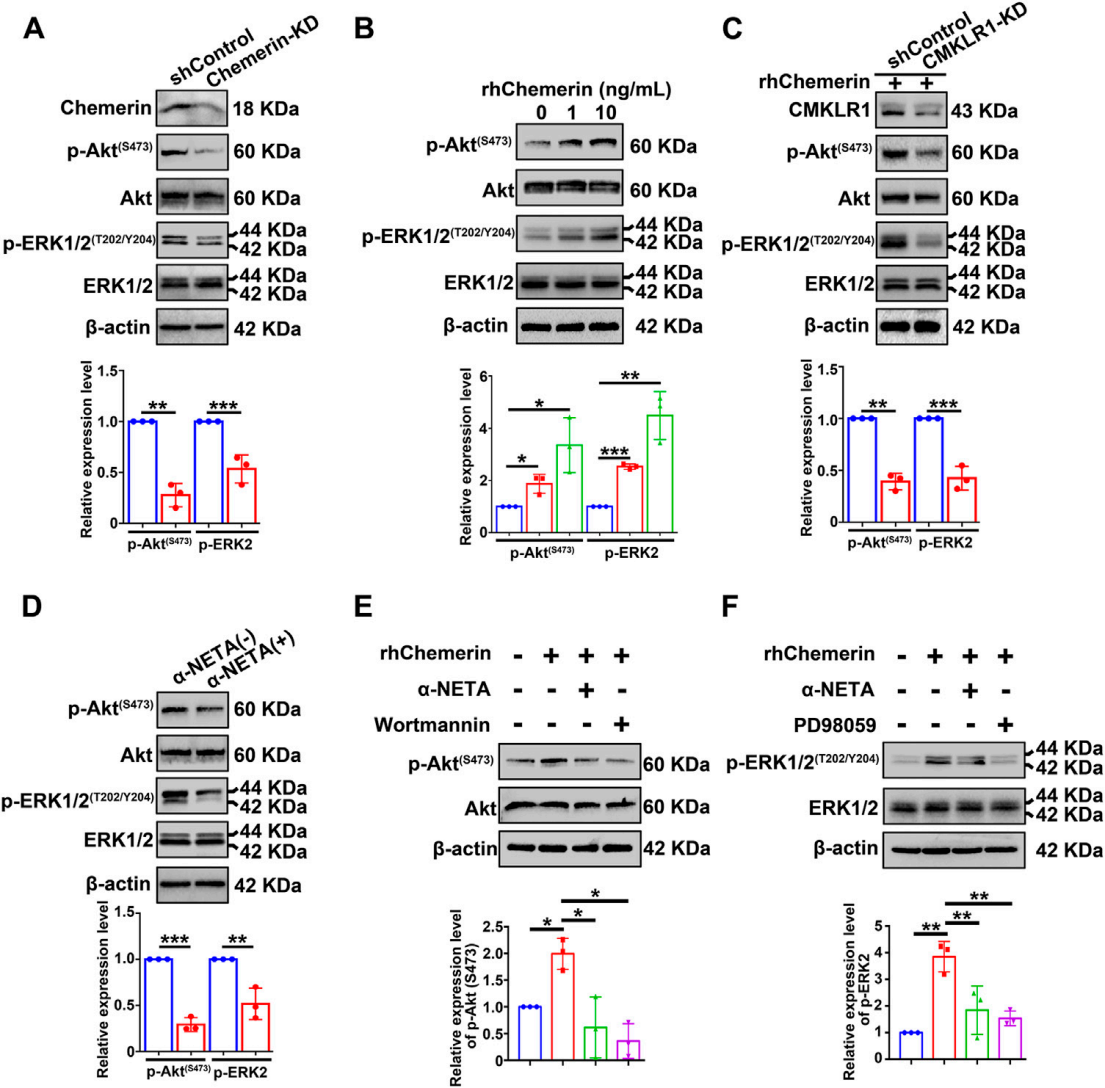
Chemerin acts on CMKLR1 to activate the PI3K/Akt and MAPK/ERK signaling pathways in hEM15A cells. **(A)** Immunoblots of chemerin, p-Akt^(S473)^, Akt, p-ERK1/2^(T202/Y204)^, ERK1/2 in shControl and chemerin-KD cells. **(B)** Immunoblots of p-Akt^(S473)^, Akt, p-ERK1/2^(T202/Y204)^, ERK1/2 in hEM15A cells treated with rh-chemerin for 5 min. **(C)** Immunoblots of CMKLR1, p-Akt^(S473)^, Akt, p-ERK1/2^(T202/Y204)^, ERK1/2 in shControl and CMKLR1-KD cells treated with rh-chemerin for 5 min. **(D)** Immunoblots of p-Akt^(S473)^, Akt, p-ERK1/2^(T202/Y204)^, ERK1/2 in hEM15A cells treated with α-NETA for 24 h. **(E)** Immunoblots of p-Akt^(S473)^ and Akt in hEM15A cells were treated with different reagents as indicated. **(F)** Immunoblots of ERK1/2^(T202/Y204)^ and ERK1/2 in hEM15A cells were treated with different reagents as indicated. β-Actin served as a loading control. Assays were performed in triplicate, and the densitometry of blots was shown. Data are shown as mean ± SD. *p < 0.05; **p < 0.01; ***p < 0.001.

In order to explore the signaling transduction mechanisms of chemerin/CMKLR1 in endometrial stromal cells, two critical signaling pathways (PI3K/Akt and MAPK/ERK) known to implicate in endometriosis establishment and development were examined. Total Akt, phospho-Akt (Ser^473^) (hereby referred to as p-Akt), total ERK1/2, and phospho-ERK1/2 (Thr^202^/Tyr^204^) (hereby referred to as p-ERK1/2) in hEM15A-shControl and chemerin-KD cells were detected by Western blot. As shown in Figure 5A, the quantitative analysis of immunoblots showed that knockdown of endogenous chemerin resulted in a marked inhibition of p-Akt and p-ERK1/2 protein levels (normalized to Akt and ERK1/2, respectively). In addition, exogenous treatment with recombinant human chemerin (rh-chemerin) (1 or 10 ng/ml for 5 min) dose-dependently increased the p-Akt and p-ERK1/2 protein amounts in hEM15A cells (Figure 5B). It is worth noting that several studies found that long-term exposure with rh-chemerin increases the PTEN expression in hepatocellular carcinoma, prostatic carcinoma, and sarcoma cell lines, thus suppressing the PI3K/Akt signaling pathway (Li et al., 2018; Rennier et al., 2020). We, therefore, asked whether this phenomenon could occur in hEM15A cells, and our results clearly showed that the PTEN protein expression was not altered after rh-chemerin (10 ng/ml) treatment for 48 h (Supplementary Figure S1C). Alternatively, the quantitative data further showed that knockdown of CMKLR1 robustly perturbed rh-chemerin-induced p-Akt and p-ERK1/2 levels suggesting that chemerin acts *via* CMKLR1 to stimulate the PI3K/Akt and MAPK/ERK signaling cascades (Figure 5C). In agreement with these findings, blockage of the chemerin/CMKLR1 axis by α-NETA significantly repressed the p-Akt and p-ERK1/2 levels in hEM15A cells (Figure 5D). α-NETA pre-treatment also hindered rh-chemerin-mediated phosphorylation of Akt and ERK1/2, and Wortmannin (inhibitor of PI3K/Akt) or PD98059 (inhibitor of MAPK/ERK) were leveraged as the positive controls (Figures 5E,F). Wortamanin combined with PD98059 treatment suppressed the expression mesenchymal markers of hEM15A cells and rh-chemerin treated hEM15A-chemerin-KD cells, and similar results were observed in the α-NETA treatment group (Supplementary Figure S1D,E). These results suggest that chemerin/CMKLR1 axis regulates the mesenchymal characteristics of endometriotic cells at least through the PI3K/Akt and MAPK signaling pathways.

### α-NETA alleviates the endometriosis progression *in vivo*

**FIGURE 6:**
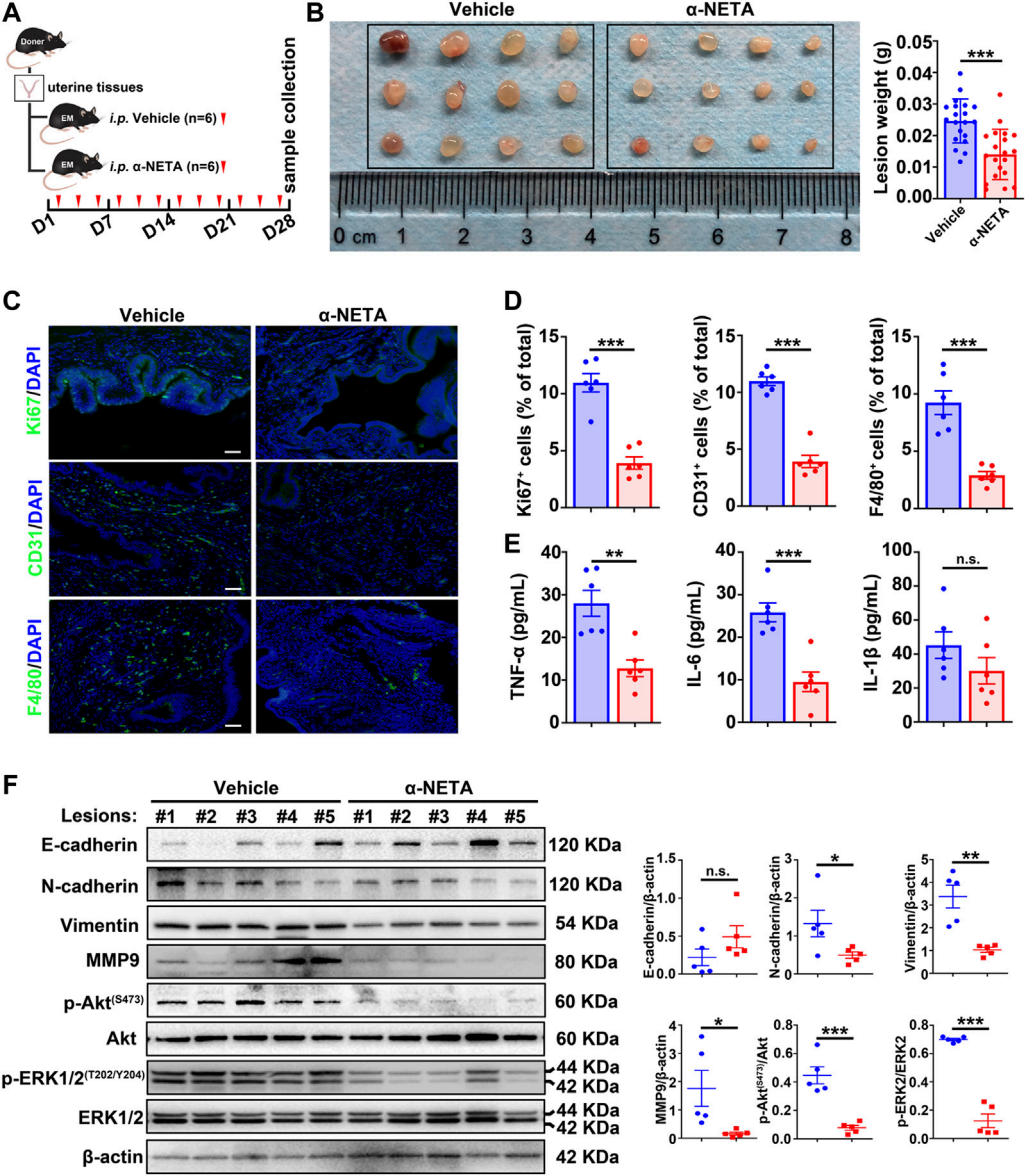
α-NETA alleviates endometriosis progression *in vivo*. **(A)** Schematic of animal experimental-2 to evaluate the effects of α-NETA on endometriosis *in vivo*. **(B)** After surgeries for 1-month, peritoneal lesions were excised, and the weight of lesions was measured. **(C)** Representative immunofluorescent images of Ki67, CD31, and F4/80 in lesions from the vehicle and α-NETA groups. Scale bars, 50 μm. Nuclei were stained with DAPI. **(D)** The quantitative data of C was shown. **(E)** TNF-α, IL-6, and IL-1β levels in peritoneal lavage fluids from vehicle (*n* = 6) and α-NETA administrated mice (*n* = 6) were detected by ELISA. **(F)** Immunoblots of E-cadherin, N-cadherin, vimentin, MMP-9, p-Akt^(S473)^, Akt, p-ERK1/2^(T202/Y204)^, ERK1/2 in lesions from vehicle (*n* = 5) and α-NETA (*n* = 5). The densitometry of blots was shown. β-Actin served as a loading control. Data are shown as mean ± SD. ns. not significant; *p < 0.05; **p < 0.01; ***p < 0.001.

Based on the aforementioned results, we reasoned that blockage of the chemerin/CMKLR1 axis by α-NETA could be a potential therapeutic approach for endometriosis treatment. To test this idea, a pre-clinical murine model was established, and vehicle or α-NETA were administered by intraperitoneal injection thrice a week for 4 weeks (Figure 6A). Results showed that α-NETA treatment significantly inhibited the size of the lesions when compared with the vehicle controls (Figure 6B) without weight loss or obvious organ damage (Supplementary Figure S1F,G). In addition, statistical analysis of IF indicated that Ki67-positive cells (a marker of cellular proliferation), CD31-positive cells (a marker of vascular endothelial cells), F4/80-positive cells (a marker of murine macrophages) and CD206-positive cells (a marker of M2-like macrophages) were dramatically reduced in lesions from the α-NETA group in comparison with the vehicle group (Figures 6C,D; Supplementary Figure S1H). Given that CMKLR1 is expressed on endometriotic cells and vascular endothelial cells in murine endometriotic lesions (Figure 1E; Supplementary Figure S1A), and F4/80-positive murine macrophages (Zabel et al., 2006), these histological results implies that blockage of chemerin/CMKLR1 axis by α-NETA restrains the endometriotic cellular proliferation, angiogenesis and immune cell chemotaxis. Meanwhile, ELISA results showed that peritoneal leverage fluids of the α-NETA group contained significantly lower TNF-α and IL-6 levels than those of vehicle controls (Figure 6E), indicating an anti-inflammatory potential of α-NETA. Furthermore, western blot results showed that α-NETA administration led to an up-regulated expression trend of E-cadherin, coupled with a significant down-regulation of N-cadherin, vimentin, and MMP9 (Figure 6F), suggesting an anti-mesenchymal effect of α-NETA. Meanwhile, α-NETA treatment simultaneously inactivated the PI3K/Akt and MAPK/ERK signaling pathways as demonstrated that the lower levels of p-Akt and p-ERK1/2 in ectopic tissues of α-NETA group relative to the vehicle group. Taken together, these data provide strong evidences that α-NETA displays an anti-endometriosis effect *in vivo*.

**FIGURE 7:**
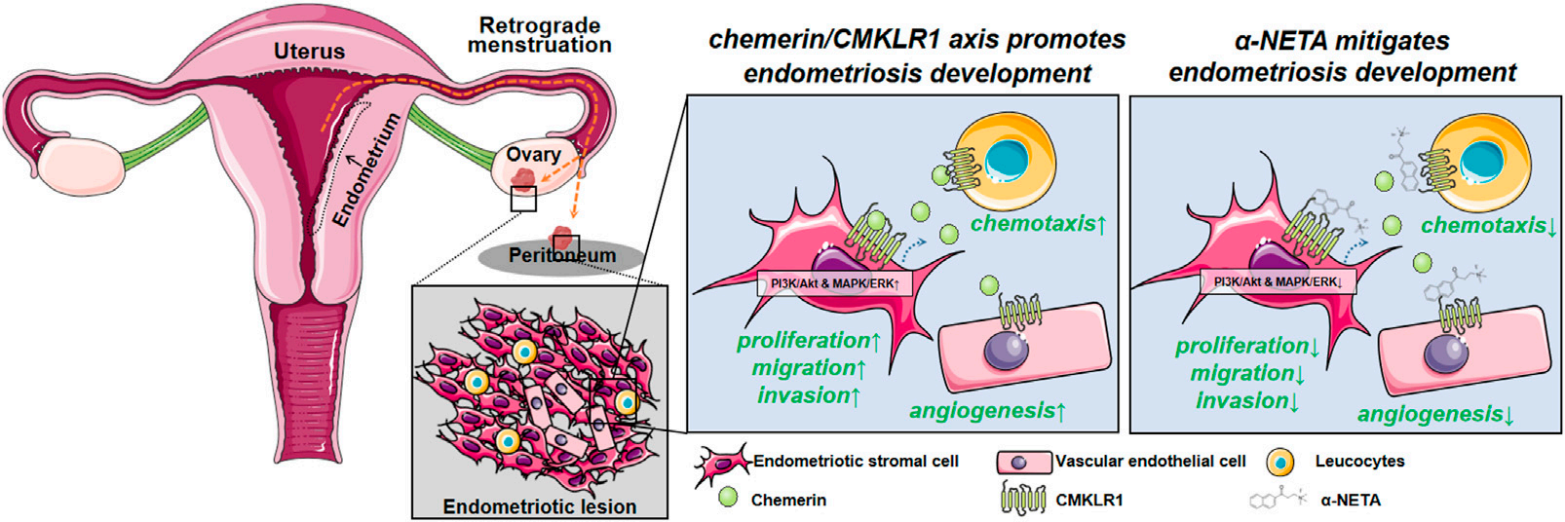
Schematic illustrating the current working model. Chemerin acts *via* CMKLR1 to regulate a series of biological events, thus promoting the endometriotic lesion establishment and development. Disrupting the chemerin/CMKLR1 axis by α-NETA alleviates the endometriosis progression.

## Discussion

In this article, we found that chemerin and CMKLR1 were aberrantly upregulated in endometriosis. Combining the data from cellular and animal models, we revealed that chemerin acts *via* CMKLR1 to activate the PI3K/Akt and MAPK/ERK signaling pathways, thus regulating the viability and mesenchymal features of endometriotic cells. Additionally, we provide evidence that CMKLR1 antagonist α-NETA exhibits profound anti-endometriosis effects (anti-growth, anti-mesenchymal features, anti-angiogenesis, and anti-inflammation) *in vivo*.

Efforts have been made for decades to find non- or minimally invasive biomarkers for the early detection and monitoring of the progression of endometriosis, unfortunately, a single reliable endometriosis biomarker is still underway. In this article, analysis of the human database found that *RARRES2* and *CMKLR1* were consistently up-regulated in peritoneal, sacrouterine ligment, rectovaginal, DIE, and ovarian lesions when compared with the eutopic controls (Figure 1A). Our experiments further confirmed that chemerin and CMKLR1 were enhanced in mouse peritoneal endometriosis (Figures 1B–F). These results are in agreement with one study published in 2015 which reported that the chemerin and CMKLR1 were markedly elevated in the ovarian endometrial lesions relative to the controls. This article also reported that peritoneal fluids of endometriosis contained higher amounts of chemerin when compared with the non-endometriosis controls. However, chemerin concentrations in serum did not differ in patients with and without endometriosis (Jin et al., 2015). Similarly, the current study found that chemerin in peritoneal leverage fluids of EM-mice was greater than those in the sham-mice (Figure 1C). Unexpectedly, chemerin in serum was significantly decreased in EM-mice relative to the sham-mice (data not shown). A possible explanation for this might be that endometriosis affects the function of liver or adipose tissue (Taylor et al., 2021), which are primary organs that contribute to the circulating chemerin. Nonetheless, according to the previous and current data, we can infer that local chemerin, but not systemic chemerin, could be considered as a potential biomarker for endometriosis. Further research should be undertaken to collect the body fluids (serum, urine, and seroperitoneum) from healthy individuals and patients to evaluate the clinical diagnostic value of chemerin.

EMT/MET is a fundamental cellular event that underlie embryonic development, tissue repair, and cancer metastasis (Kalluri and Weinberg, 2009). EMT is generally characterized by the loss of the epithelial features and gain of traits of mesenchymal cells. The induction of EMT is triggered by a variety of factors/signaling pathways, and these EMT-inducers promote the expression of EMT-transcription factors, which further repress the epithelial-related biomarkers (E-cadherin, etc.) along with activating the mesenchymal-related biomarkers (N-cadherin, vimentin, MMPs, etc.) (Dongre and Weinberg, 2019). EMT is an increasingly recognized phenomenon in endometriotic lesions (Zondervan et al., 2018). The immunohistochemical and bioinformatic analysis found that EMT-related biomarkers are significantly altered in ectopic endometrial tissues relative to the eutopic tissues (Chen et al., 2020; Konrad et al., 2020). More importantly, EMT serves as a critical biological step for endometriosis progression, because EMT results in a more aggressive capacity of the endometriotic cells (increased MMPs), leading to progesterone resistance (down-regulation of progesterone receptor) and triggering the formation of fibrosis (Zhang et al., 2016; Zondervan et al., 2018; Ma et al., 2021). As reviewed by Yan-Meng Yang and Wan-Xi Yang, hypoxia and estrogen initiate the EMT through different pathways (TGF-β/Smad and Wnt/β-catenin, etc.) in endometriosis (Yang and Yang, 2017). Herein, we obtained new evidence that inhibition of the chemerin/CMKLR1 axis by genetical or pharmacologic approaches reverses the mesenchymal features of hEM15A cells (Figures 2E–G, 3D–F, 4E,F) and murine endometriotic tissues (Figure 6F). In addition, chemerin was also found to promote EMT, migration, and invasion in oral squamous cell carcinoma (OSCC) cell lines (Lu et al., 2019). However, Hyungkeun Kim *et al* reported the opposite results in breast cancer cell lines (Kim et al., 2020), suggesting that the role of chemerin in EMT/MET is cell-context specific and cell-type dependent. Further research should be undertaken to investigate the basic mechanism underlying chemerin-induced EMT/MET. In addition, given that EMT/MET is essential in endometrial regeneration and decidualization (Owusu-Akyaw et al., 2019), it will be of great interest to study the role of the chemerin/CMKLR1 axis in endometrial physiological functions.

Chemerin was initially identified as a chemoattractant, acts through CMKLR1 expressed on certain leucocyte populations, controlling chemotaxis towards the area of inflammation (Wittamer et al., 2003). Mounting evidence suggests that chemerin/CMKLR1-induced immune cell chemotaxis participates in various physiological processes and diseases. For example, during early pregnancy, trophoblasts and decidua stromal cells secrete abundant chemerin to aggregate NK cells into the placenta to further ensure successful pregnancy (Carlino et al., 2012). Apart from its role in immunity, previous studies evidenced that the chemerin/CMKLR1 axis is angiogenic *in vitro* and *in vivo* (Bozaoglu et al., 2010; Kaur et al., 2010; Nakamura et al., 2018). In consideration of chronic inflammation and neovascularization are two key processes for endometriosis development, we hypothesized that locally enhanced chemerin might contribute to these two vital pathological events. Through a pharmacological approach, α-NETA administration significantly suppressed the CD31-positive and F4/80-positive cells in the murine-endometriosis lesions compared with the vehicle controls (Figures 6D,E). α-NETA also inhibited the infiltrating CD206-positive M2-like macrophages (Supplementary Figure S1H), which were reported to participate in the angiogenesis (Ono et al., 2021) and fibrogenesis (Duan et al., 2018) of endometriotic lesions in mice. Due to the reasons that CMKLR1 is expressed on vascular endothelial cells in murine endometriotic lesions (Supplementary Figure S1A), and F4/80-positive murine macrophages (Zabel et al., 2006). These results imply that the chemerin/CMKLR1 axis seems to contributes to angiogenesis and inflammations during endometriosis progression, and targeting this axis is plausible for endometriosis treatment.

Currently, three types of CMKLR1 antagonists including polypeptides (C15), nanobodies (CA4910 and CA5183), and small molecule compounds (CCX832 and α-NETA) were developed (Kennedy and Davenport, 2018). CMKLR1 antagonists display a promising therapeutic effect in several animals disease models such as the myocardial infarction model (C15), type 2 diabetes model (CCX832), multiple sclerosis model (α-NETA), neuroblastoma xenograft model (α-NETA), and preeclampsia model (α-NETA) (Cash et al., 2013; Graham et al., 2014; Tümmler et al., 2017; Neves et al., 2018; Ji et al., 2021). In this work, we found that α-NETA inhibited the growth of hEM15A cells and hEM15A-speriods (Figures 4A–D). α-NETA treatment resulted in MET, weakened motility, and invasion ability of hEM15A cells (Figures 4E,F). Most importantly, our pre-clinical animal model results revealed that α-NETA mitigated the endometriosis progression (anti-growth, anti-mesenchymal features, anti-angiogenesis, and anti-inflammation) without weight loss and obvious organ damage (Figure 6; Supplementary Figure S1F,G). These data raise the possibility that α-NETA serves as a potential adjuvant drug for endometriosis therapy. α-NETA appears to exhibit reliable security *in vitro* and *in vivo* (Kumar et al., 2019). Given that oral administration of a high dosage of α-NETA (300 mg/kg) daily for 14 days did not affect the mouse body weight or gross morphological appearance of vital organs (Kumar et al., 2019), we are interested to evaluate the effects of oral gavage with α-NETA on endometriosis development, pain performance and fertility status of EM-mice in future.

In endometriosis, multiple abnormal signaling cascades were responsible for survival, proliferation, EMT, migration, invasion, and anti-apoptosis of endometriotic cells. Among these pathways, PI3K/Akt and MAPK/ERK pathways were widely examined, and the application of inhibitors (Sorafenib, Vemurafenib, U0126, MK2206, WIN 55212–2) was shown to have profound therapeutic efficacy *in vitro* and *in vivo*, despite they have certain side-effects (Hung et al., 2021). In the endometriotic milieu, estradiol, inflammatory factors (TNF-α, IL-6, IL-1β, etc.) or growth factors (EGF, etc.) were enhanced, and these mediators activate the PI3K/Akt and MAPK/ERK pathways in endometriotic cells. In this article, results in Figures 5A,B showed that exogenous or endogenous chemerin impacts the phosphorylation of Akt and ERK1/2 in hEM15A cells. Moreover, results in Figures 5C–F provide strong evidence that genetical and pharmaceutical inhibition of CMKLR1 hindered rh-chemerin-activated p-Akt and p-ERK1/2 in hEM15A cells. Most importantly, α-NETA simultaneously restrained the p-Akt and p-ERK1/2 levels in hEM15A cells and murine-endometriosis lesions (Figure 5D and Figure 6F), which could further impair the biological functions of endometriotic cells (Supplementary Figure S1D,E). Supported by Banu SK and coworker’s proposal that dual inhibition of PI3K/Akt and MAPK/ERK is indispensable for the suppression of endometriosis establishment and progression (Arosh and Banu, 2019; Arosh et al., 2021), we provide molecular insights into the therapeutic effects of targeting chemerin/CMKLR1 axis by α-NETA in endometriosis.

Taken together, using a combination of bioinformational, genetical, pharmacological, and histological analysis approaches, we show here that the chemerin/CMKLR1 axis is crucial for endometriosis development. We believe that these findings will help in a better understanding of the pathological mechanisms in endometriosis, and may allow for the development of clinical therapeutic drugs.

## Materials and methods

### Antibodies and reagents

Antibodies against Chemerin (10216-1-AP, Proteintech), CMKLR1 (sc-398769, Santa Cruz Biotechnology), E-cadherin (#3195, Cell Signaling Technology), N-cadherin (#14215, Cell Signaling Technology), Vimentin (ab92547, Abcam), MMP9 (ab38898, Abcam), PTEN (22034-1-AP, Proteintech), Akt (#4691, Cell Signaling Technology), phospho-Akt (Ser^473^) (#4060, Cell Signaling Technology), ERK1/2 (#4695, Cell Signaling Technology), phospho-ERK1/2 (Thr^202^/Tyr^204^) (#4370, Cell Signaling Technology), GAPDH (ab9484, Abcam), β-actin (#4970, Cell Signaling Technology), Ki67 (ab15580, Abcam), CD31 (ab222783, Abcam), F4/80 (ab6640, Abcam) and CD206 (18704-1-AP, Proteintech) were utilized for this study. Recombinant human chemerin protein (2324-CM) was obtained from R&D Systems. α-NETA (31059-54-8) was obtained from Santa Cruz Biotechnology. Wortmannin (HY-10197), PD98059 (HY-12028), and Sulfobutylether-β-Cyclodextrin (SBE-β-CD) (HY-17031) were purchased from MCE. Matrigel (Growth Factor Reduced, Phenol-Red Free) (356238) was purchased from Corning.

### Animals and experimental design

All animal experimental procedures were performed according to protocols approved by the Institutional Animal Care and Use Committee (IACUC) at SIAT (#SIAT-IACUC-200313-YYS-YM-A1105). All mice used in this study were maintained under controlled conditions with a light/dark cycle of 12/12 h and had access to food and water *ad libitum*. 7-week-old virgin female C57BL/6J mice were directly purchased from the GemPharmatech (Guangdong, China), and bred in specific pathogen-free facilities at SIAT.

A syngeneic surgical model of endometriosis was established according to the protocol published online (Escudero-Lara et al., 2020). Briefly, mice at diestrus were selected as the donors, and their uterine and abdominal fat tissues were excised. One uterine horn was excised and longitudinally opened with scissors. Uterine horn or fat tissues were dissociated into four pieces (2 mm × 2 mm). Next, the uterine fragments were sutured into the abdominal wall (two on each side of the incision with the endometrium facing the abdominal wall) to generate the peritoneal endometriosis-like lesions. Fat tissues were sutured into the abdominal wall (two on each side of the incision) to serve as the sham control. Four weeks after surgeries, euthanize the recipient mice by cervical dislocation, and the samples were collected and analyzed. Two animal experiments with different purposes were performed in this study, and each experiment was carried out three times.

Experimental-1 was designed to examine the chemerin and CMKLR1 expression in a mouse-EM model. A total of 15 mice were used, three in diestrus were selected as donors to provide the fat and uterine tissues. 12 recipients were randomly divided into two groups: six mice received with fat tissues were defined as the “sham”, and six mice received with uterine fragments were defined as the “EM”. (Figure 1B).

Experimental-2 was designed to evaluate the effects of α-NETA on endometriosis *in vivo*. A total of 18 mice were used. Six in diestrus were selected as donors to provide the uterine tissues. 12 surgically induced EM mice were randomly divided into two groups: “vehicle” and “α-NETA”. α-NETA was formulated in a 10% SBE-β-CD vehicle for *in vivo* dosing. α-NETA (3 mg/kg, 100 μl) or vehicle (10% SBE-β-CD in saline, 100 μl) were administered by intraperitoneal injection thrice a week for 4 weeks. The body weight of mice was recorded on the day of dosing (Figure 6A).

### Cell culture

hEM15A and Ishikawa cell lines were purchased from BeNa Culture Collection. Ishikawa cells were cultured in DMEM/Basic (1×) supplemented with 10% fetal bovine serum (FBS) and 1% penicillin-streptomycin. hEM15A cells were cultured in DMEM/F-12 supplemented with 10% fetal bovine serum (FBS) and 1% penicillin-streptomycin. All cell lines were characterized as *mycoplasma* negative using the MycoBlue *Mycoplasma* Detector (D101-02, Vazyme) according to the manufacturer’s instructions. Cells were cultured in a humidified atmosphere containing 5% CO_2_ at 37°C. The medium was renewed every 2–3 days. To generate stable genetically modified hEM15A cell lines, empty vector, shRNA targeting *RARRES2* (stem sequence: AGC CCT TCC CAG CTG GAA TATT) or *CMKLR1* (stem sequence: AGG TGA TGA ATA CCC TGA TTAT) were transiently transfected in hEM15A cells by using Lipofectamine 2000 reagent (Invitrogen). Two days later, 2.5 μg/ml puromycin was added to the culture medium, and single-cell clones were further screened, the knockdown efficiency was validated by qPCR and western blot.

### Quantitative real-time PCR

Cells were treated with RNAiso Plus reagent (Takara) for RNA extraction, and the PrimeScript™ RT Master Mix (RR036A, Takara) was used to synthesize cDNA. TB Green^®^ Premix Ex Taq™ (Tli RNaseH Plus) (RR420L, Takara) was used for qPCR. The primers were as follows: *RARRES2*: 5-TGG AAG AAA CCC GAG TGC AAA-3′ (forward), 5′-AGA ACT TGG GTC TCT ATG GGG -3′ (reverse); *CMKLR1*: 5′-ATG GAC TAC CAC TGG GTT TTC GGG-3′ (forward), 5′-GAA GAC GAG AGA TGG GGA ACT CAA G-3′ (reverse); *ACTB*: 5′-AGC GAG CAT CCC CCA AAG TT -3′ (forward), 5′-GGG CAC GAA GGC TCA TCA TT -3′ (reverse). The reactions were performed using the LightCycler^®^ 96 Real-time PCR System (Roche, Switzerland). Quantified data were normalized to those of *ACTB*, and the relative quantity was calculated using the 2^−ΔΔCT^ method.

### Protein isolation and western blot

Proteins were extracted by M-PER™ Mammalian Protein Extraction Reagent (78501, Thermo Fisher), and the concentrations were quantified by Pierce™ BCA (23227, Thermo Fisher). Equal proteins were loaded onto 12% SDS-PAGE gels and then were transferred onto a nitrocellulose membrane (Merck Millipore). After blocking with 5% non-fat dry milk for 1.5 h, the membranes were incubated at 4°C overnight with the primary antibody; Next, the membranes were incubated with HRP-labeled goat anti-mouse or goat anti-rabbit IgG for 1 h. An enhanced chemiluminescence (ECL) detection system (Bio-Rad, United States) was used to visualize immunoreactive bands. Except for western blots including multiple tissue samples, each western blot experiment was repeated three times with independent sample sets.

### ELISA

The conditional medium of hEM15A and primary human endometrial stromal cells were collected to detect the human chemerin levels. Peritoneal leverage fluids of a sham- or EM-mice were collected to detect the mouse chemerin levels. ELISA kits for human chemerin (DCHM00) and mouse chemerin (MCHM00) were purchased from R&D Systems. Peritoneal leverage fluids of vehicle- or α-NETA administrated mice were collected to detect the mouse TNF-α, IL-6, and IL-1β levels by utilizing the ELISA kits purchased from BioLegend (#430904, #431304 and #432604).

### Immunofluorescence and immunohistochemistry

Mouse tissues were collected and fixed in 4% paraformaldehyde. Paraffin-embedded and OCT-embedded tissues were sectioned onto charge slides. For immunohistochemistry, after antigen retrieval, dewaxed hydrated paraffin-embedded tissue sections were immersed in 3% H_2_O_2_ and 100% methanol for 30 min at room temperature to quench endogenous peroxidase, and then the sections were blocked with blocking buffer (10% normal serum with 1% BSA in TBS) for 2 h at room temperature, and incubated with the first antibody at 4°C overnight. Next, the sections were incubated with HRP conjugate second antibody for 1h at room temperature followed by detection and counterstain. For immunofluorescence, the sections were blocked and incubated with the first antibody at 4°C overnight. Next, the sections were incubated with Alexa Fluor^®^ 488-labeled goat anti-rabbit IgG or Alexa Fluor^®^ 488-labeled goat anti-rat IgG (Abcam) for 1 h followed by counterstaining with DAPI.

### Cell viability assay

hEM15A cells were seeded at 2000 cells/well in 96-well plates, with five wells used for each group. Cell viability was evaluated over 4 days using Cell Counting Kit-8 (CCK-8) (Beyotime Biotechnology, #C0038). 10 μl CCK-8 plus 90 μl DMEM/F12 medium without FBS (detection buffer) was added to each well and incubated at 37°C for 1.5 h. The absorbance at 450 nm was measured in each well by using a microplate reader (Thermo Fisher Scientific). For the cytotoxicity assay, hEM15A cells were seeded at 4000 cells/well in 96-well plates, and α-NETA (0, 1.25, 2.5, 5, 10, 20, 40, 80, 160, 320 μm) was added to the wells for 24 h followed by replacement with detection buffer, and the absorbance at 450 nm was measured with the microplate reader. IC_50_ (half maximal inhibitory concentration) of α-NETA was calculated by GraphPad Prism 9.0 (La Jolla, CA, United States).

### Clone formation assay

hEM15A cells were seeded at 1000 cells/well in a 6-well plate. Following cell adherence, the culture systems were treated in the presence or absence of 25 μm α-NETA. After 2 weeks, plates were fixed with methanol and stained with crystal violet (0.5% w/v). The colony is defined to consist of at least 50 cells, and the colony numbers were counted.

### Transwell assay

Cell migration and invasion were determined using a 24-wells Transwell chamber (8 μm pores) coated without or with Matrigel, respectively. 50 μl Matrigel diluted with the precooled serum-free DMEM/F12 (1/10) was added to the upper chamber of the Transwell and incubated at 37°C for 30 min 200 μl serum-free DMEM/F-12 medium containing 1 × 10^5^ hEM15A cells were transferred to the top chambers. 1 ml 10% FBS DMEM/F-12 medium was added in the 24-wells as the chemotactic agents. After incubating for 24 h (migration) or 48 h (invasion), cells on the upper side of the inserts were removed by a cotton swab. The cells on the bottom of the inserts were fixed in methanol and stained with crystal violet. The number of migrated or invaded cells was counted under a light microscope in four random fields. Three independent experiments were performed.

### hEM15A-spheroids preparation, calcein AM, and EthD-1 staining

To generate the hEM15A cell-derived spheroids, hEM15A cells were seeded at 2000 cells/well in 96-well Clear Flat Bottom Ultra-Low Attachment Microplate (3474, Corning). Adding 2.5% Matrigel in the culture system (10%FBS DMEM/F-12 medium) was indispensable to form hEM15A-spheroids. α-NETA (0, 25, 50 μm) was added to the plates for 48 h, and the diameter of spheroids was recorded. Live and Dead™ Viability/Cytotoxicity Assay Kit for Animal Cells (Calcein AM, EthD-1) was purchased from Yu Heng Bio (L6023). hEM15A-spheroids were stained with Calcein AM and EthD-1 according to the manufacturer’s experimental procedure and observed under an inverted fluorescence microscope. Three independent experiments were performed.

### Statistical analysis

All statistical analyses were performed using GraphPad Prism 9.0 (La Jolla, CA, United States). Data are presented as mean ± SD. Statistical analysis between two groups was performed using Student’s t-test, and analysis between multiple groups was conducted by one-way analysis of variance (ANOVA).

## Data Availability

The original contributions presented in the study are included in the article/Supplementary Material, further inquiries can be directed to the corresponding authors.
